# Retrospective study of prognosis of patients with multiple colorectal carcinomas: synchronous versus metachronous makes the difference

**DOI:** 10.1007/s00384-021-03926-6

**Published:** 2021-04-14

**Authors:** Christoph Barz, Christian Stöss, Philipp-Alexander Neumann, Dirk Wilhelm, Klaus-Peter Janssen, Helmut Friess, Ulrich Nitsche

**Affiliations:** Klinikum rechts der Isar, Department of Surgery, School of Medicine, Technical University of Munich, Ismaninger Str. 22, 81675 Munich, Germany

**Keywords:** Colorectal cancer, Multiple cancers, Synchronous cancers, Prognosis

## Abstract

**Purpose:**

Little is known about difference between synchronous colorectal cancer (SCRC) and metachronous colorectal cancer (MCRC) despite the relevance for this selected patient group. The aim of this retrospective review was to analyze patients with SCRC and MCRC.

**Methods:**

All patients who underwent surgery for SCRC and MCRC between 1982 and 2019 were included in this retrospective analysis of our tertiary referral center. Clinical, histological, and molecular genetic characteristics were analyzed. The primary endpoint was cause-specific survival, evaluated by the Kaplan-Meier method. Secondary endpoints were recurrence-free survival and the identification of prognostic factors.

**Results:**

Overall, 3714 patients were included in this analysis. Of those, 3506 (94.4%) had a primary unifocal colorectal cancer (PCRC), 103 (2.7%) had SCRC, and 105 (2.8%) had MCRC. SCRC occurred more frequently in elderly (*p*=0.009) and in male patients (*p*=0.027). There were no differences concerning tumor stages or grading. Patients with SCRC did not show altered recurrence or survival rates, as compared to unifocal tumors. However, MCRC had a lower rate of recurrence, compared to PCRC (24% vs. 41%, *p*=0.002) and a lower rate of cause-specific death (13% vs. 37%, *p*<0.001). Five-year cause-specific survival rates were 63±1% for PCRC, 62±6% for SCRC (*p*=0.588), and 88±4% for MCRC (*p*<0.001). Multivariable analysis revealed that MCRC were an independent favorable prognostic parameter regarding case-specific survival.

**Conclusion:**

Patients with SCRC seem to not have a worse prognosis compared to patients with PCRC. Noteworthy, patients with MCRC showed better survival rates in this retrospective analysis.

**Supplementary Information:**

The online version contains supplementary material available at 10.1007/s00384-021-03926-6.

## Introduction

Colorectal cancer (CRC) is the third most common cancer in the western world [1]. By far most of these patients present with a primary unifocal adenocarcinoma of the colorectum (PCRC), which enables treatment according to established and validated guidelines [2]. However, in about 2–8% of all patients, synchronous multiple CRC (SCRC) are diagnosed at the time of diagnosis or during postoperative pathologic examination [3–5]. Further, albeit it may be sometimes difficult to discriminate from a recurrence of the initial tumor, about 1.6–2.4% of patients who undergo a complete resection (R0) of their primary colorectal cancer will develop a second, metachronous colorectal cancer (MCRC) during follow-up. MCRC was described to have a higher age-corrected incidence than compared to healthy controls who did not have colorectal cancer before [6–9]. Due to their relative rare occurrence, available clinical data on SCRC and MCRC is limited. Some authors postulated that sporadic (non-HNPCC and non-APC) SCRCs have a high molecular inter-tumoral homogeneity and may be associated with the MSI-/BRAF-pathway, leading to reduced overall survival [3, 10]. Others describe SCRC to have essentially different driver mutations in the tumor-associated genes KRAS, BRAF, and p53—and a comparable or even slightly better prognosis than classical unifocal tumors [5, 11]. For MCRC, in the limited literature available, the mean time to occurrence is estimated approximately 4 years after the initial colorectal cancer [2]. This is considerably later compared to the “classical” local/regional tumor recurrence, of which 80% of all cases occur within the first 2 years after initial treatment [2]. Finally, patients with SCRC seem to have a 3- to 6-fold higher chance for developing MCRC [6, 7, 12].

Still many uncertainties exist for SCRC or MCRC. It is not clear whether these patients bear specific histological characteristics, accompanied by a distinct recurrence and survival profile. Here, we sought to analyze the impact on multiple cancers within identical patients on oncological outcomes and disease progression.

## Materials and methods

### Study population

Since 1982, all patients undergoing surgery for colorectal cancer at the Department of Surgery, Klinikum rechts der Isar, Technische Universität München, Munich, Germany, are scheduled for periodic follow-up either at our interdisciplinary outpatient tumor center or outside of the hospital and according to the recommendations of the German Cancer Society. All patient data are prospectively entered to a data base. The ethics committee of the Klinikum rechts der Isar approved the study (no. 1926/7). Informed written consent was obtained from all patients prior to the collection of data. This observational cohort study was drafted in accordance with the STROBE statement (http://www.strobe-statement.org).

For the present analysis, consecutive complete datasets of patients with surgery for PCRC, SCRC, or MCRC were extracted. In order to report on consecutive data sets and to avoid selection bias, patients with missing follow-up data (Table 1) were not excluded. Patients with proven or suspected familial adenomatous polyposis (FAP) or hereditary nonpolyposis colorectal cancer (HNPCC), patients with proven or suspected tumor recurrence (see definitions below), and patients with an uncertain past medical history regarding prior malignancies were not included. The latest date of inclusion and follow-up was July 2019. Clinical histopathological, molecular genetic, and follow-up data (recurrence/survival) of patients with PCRC were compared to those of patients with SCRC and MCRC.

**Table 1:** Characteristics of the complete patient cohort

	*n*=3714	%	Effect on cause-specific survival
Tumor type			
Primary unifocal CRC (PCRC)	3506	94	HR 1
Synchronous multiple CRCs (SCRC)	103	3	*p*=0.057 (HR 1.38; 95% CI 0.99–1.91)
Metachronous CRC (MCRC)	105	3	*p*<0.001 (HR 0.27; 95% CI 0.14–0.52)
Patient age			
Years (median)	65	100	*p*=0.663 (HR 1.00; 95% CI 0.99–1.00)
Gender			
Men	2135	57	HR 1
Women	1579	43	*p*=0.088 (HR 1.11; 95% CI 0.99–1.25)
Colon versus Rectum (main tumor)			
Colon	2203	59	HR 1
Rectum	1511	41	*p*=0.320 (HR 1.06; 95% CI 0.94–1.20)
Localization (all tumors)			
Multiple Segments	30	1	HR 1
Right hemicolon	1134	31	*p*=0.081 (HR 0,59; 95% CI 0.32–1.07)
Left hemicolon	1013	27	*p*=0.049 (HR 0.55; 95% CI 0.30–0.99)
Rectum	1491	41	*p*=0.101 (HR 0.61; 95% CI 0.33–1.10)
Missing	46		
Preoperative ileus			
No ileus	3175	89	HR 1
Subileus	200	6	*p*<0.001 (HR 2.16; 95% CI 1.72–2.70)
Manifest ileus	178	5	*p*<0.001 (HR 2.40; 95% CI 1.91–3.03)
Missing	161		
Concomitant diseases			
No	1547	45	HR 1
Yes	1891	55	*p*=0.353 (HR 1.14; 95% CI 0.87–1.50)
Missing	276		
**Tumor size**			
Centimeter (median)	4.0	100	*p*<0.001 (HR 1.14; 95% CI 1.11–1.16)
Missing	286		
Multivisceral surgery			
No	2814	80	HR 1
Yes	691	20	*p*<0.001 (HR 1.65; 95% CI 1.44–1.90)
Missing	209		
Tumor status			
T0	65	2	HR 1
Tis	23	1	*p*<0.001 (HR 0.89; 95% CI 0.04–0.20)
T1	349	10	*p*=0.003 (HR 0.05; 95% CI 0.01–0.45)
T2	623	18	*p*<0.001 (HR 0.05; 95% CI 0.03–0.08)
T3	1849	52	*p*<0.001 (HR 0.12; 95% CI 0.10–0.16)
T4	616	17	*p*<0.001 (HR 0.43; 95% CI 0.38–0.49)
Missing	189		
Nodal status			
N0	1943	56	HR 1
N1	786	22	*p*<0.001 (HR 3.26; 95% CI 2.77–3.85)
N2	763	22	*p*<0.001 (HR 9.15; 95% CI 7.88–10.64)
Missing	222		
Metastasis status			
M0	2605	76	HR 1
M1	834	24	*p*<0.001 (HR 10.7; 95% CI 9.47–12.16)
Missing	275		
Stage			
0/Tis	71	2	HR 1
I	753	22	*p*=0.596 (HR 0.78; 95% CI 0.31–1.96)
II	900	26	*p*=0.066 (HR 2.31; 95% CI 0.95–5.63)
III	860	25	*p*<0.001 (HR 5.99; 95% CI 2.48–14.50)
IV	834	25	*p*<0.001 (HR 31.09; 95% CI 12.88–75.06)
Missing	296		
Grading			
G1/2	2381	69	HR 1
G3/4	1064	31	*p*<0.001 (HR 2.20; 95% CI 1.95–2.48)
Missing	269		
Lymphatic invasion			
L0	2599	74	HR 1
L1	896	26	*p*<0.001 (HR 3.28; 95% CI 2.90–3.70)
Missing	219		
Vascular invasion			
V0	3187	91	HR 1
V1	304	9	*p*<0.001 (HR 3.96; 95% CI 3.36–4.67)
Missing	223		
R status			
R0	2781	79	HR 1
R1	107	3	*p*<0.001 (HR 5.94; 95% CI 4.53–7.79)
R2	627	18	*p*<0.001 (HR 15.27; 95% CI 13.33–17.50)
Missing/Rx	199		
Microsatellite status			
MSS	270	75	HR 1
MSI-H	91	25	*p*=0.011 (HR 0.34; 95% CI 0.15–0.78)
Not performed	3353		
KRAS status			
Wildtype	206	65	HR 1
Mutated	111	35	*p*=0.011 (HR 1.93; 95% CI 1.17–3.19)
Not performed	3397		
BRAF status			
Wild type	243	86	HR 1
Mutated	41	14	*p*=0.110 (HR 0.44; 95% CI 0.16–1.21)
Not performed	3430		
Tumor perforation			
No	2544	94	HR 1
Yes	171	6	*p*<0.001 (HR 2.61; 95% CI 2.02–3.38)
Missing	999		
Tumor histology			
Classical adenocarcinoma	3079	86	HR 1
Mucinous adenocarcinoma	382	11	*p*=0.011 (HR 1.27; 95% CI 1.06–1.53)
Signet-ring cell carcinoma	32	1	*p*<0.001 (HR 3.23; 95% CI 2.07–5.03)
Other types	65	2	*p*<0.001 (HR 2.34; 95% CI 1.62–3.39)
Missing	156		
CEA (ng/ml, median)			
ng/ml (Median)	4.0	100	*p*<0.001 (HR 1.00; 95% CI 1.00–>1.00)
Not performed	2491		
Tumor recurrence			
No	1874	59	HR 1
Yes	1304	41	*p*<0.001 (HR 299.67; 95% CI 169.42–530.03)
Lost of follow-up	536		
Survival status			
Alive	1457	47	n.a.
Death cancer-related	1137	37	n.a.
Death postoperative	112	3	n.a.
Death other causes	397	13	n.a.
Lost of follow-up	611		

Absolute numbers of patients together with percentages are displayed, if not indicated otherwise. The right column shows the prognostic relevance for each parameter, regarding case-specific survival upon univariable analysis. Concomitant diseases included any kind of cardiovascular, renal, pulmonary, or other relevant underlying medical conditions. *HR* hazard ratio, *95% CI* 95% confidence interval

### Definitions of synchronous and metachronous cancer

In the absence of generally accepted definitions, we considered patients to have SCRC if more than one histologically proven malignant lesion was present within the colon and/or rectum at the index assessment, clearly separated by healthy mucosa [4, 5]. The exact time point of diagnosis might vary between the preoperative (colonoscopy), intraoperative, or postoperative (histological workup of the resected specimen) time point. A complete colonoscopy was sought for all patients, which may have included a postoperative investigation of the remaining colon after resection of an obstructing tumor of the rectum or left colon. We considered patients to have MCRC, if they were diagnosed free of tumor for at least 12 months after initial treatment for colorectal cancer, and then during follow-up had a new, histological proven malignant lesion within the colon and/or rectum. The new lesion had to be divided from any anastomotic region of prior treatments by healthy mucosa and had to be unambiguously an exulcerating epithelial carcinoma. By this, the risk of overestimating the MCRC rate due to local or regional (nodal, etc.) tumor recurrences of prior occurrences was reduced to the minimum.

### Statistical analysis

Statistical evaluation was performed using IBM® SPSS® statistics Version 24 (SPSS Inc., IBM Corporation Software Group, Somers, NY, USA). The distribution of nominal or ordinal scaled variables was compared using Pearson’s chi-square test. Cardinal variables were tested for normal distribution by the Kolmogorov-Smirnov test. Explorative comparison of independent groups was performed by the t test for normal distribution and the Mann-Whitney *U* test (two groups) or the Kruskal-Wallis test (more than two groups) for non-normal distribution. All statistical tests were performed two-sided, and *p*-values less than 0.05 were considered statistically significant. No correction of *p*-values was applied to adjust for multiple testing. However, results of all statistical tests being conducted were thoroughly reported, so that an informal adjustment of *p*-values can be performed while reviewing the data [13]. Multivariable analysis of binary outcome data was assessed by logistic regression. Time-dependent survival probabilities were estimated with the Kaplan-Meier method, and the log rank (Mantel-Cox) test was used to compare independent subgroups. Cause-specific survival was used as the primary outcome parameter. Cause-specific survival is equivalent to disease-specific survival in relation to the initial malignant disease and considers only tumor-related deaths of the reported malignancy as events. It reflects the intrinsic biology of the colorectal cancer under investigation more precisely than, e.g., cancer-specific survival, which includes deaths due to any kind of cancer [14]. To investigate the effect on survival of multivariable relationships among covariates, Cox proportional hazard models were applied. Cause-specific survival times as well as estimated hazard ratios (HRs) were calculated and reported as 95% confidence intervals (95% CIs) [15].

## Results

### Study population

Between January 1982 and July 2019, 4367 consecutive patients underwent oncological resection of their histologically proven colorectal cancer(s). Of those, 619 patients had to be excluded because of another, non-colorectal carcinoma before the index operation or during follow-up, in order to minimize bias. Another 32 patients with histologically not further specified colorectal cancer were excluded. Finally, two patients who each had both, SCRC and MCRC, were omitted from the overall analysis, but are described in more detail in the results section. Thus, 3714 resected patients were finally analyzed. Of those 3714 patients, 3506 patients had PCRC (94.4%), 103 had SCRC (2.8%), and 105 had MCRC (2.8%). The median age of all patients was 65 years (range: 15 to 97 years). There were more men (*n*=2135) than women (*n*=1579) in the cohort. The tumors were located within the right hemicolon between cecum and transverse colon in 1134 patients (31%), within the left hemicolon between descending colon and sigmoid in 1013 patients (27%), within the rectum in 1491 patients (41%), and in more than one of the above mentioned colorectal segments in 30 patients (in the case of SCRC; 1% of all patients). For 46 patients, the exact allocation of the tumors to an anatomical segment was not possible. All patients underwent resection of their colorectal tumors. For all 2584 patients without metastasis (stage 0/Tis to III), R0 resection was achieved in 96% (*n*=2481). For the 834 patients who presented with distant organ metastasis or peritoneal carcinomatosis (stage IV), an R0 resection was achieved in 19% (n=160). The median follow-up was 97 months, with no differences for PCRC, SCRC, and MCRC. See Table 1 for further characteristics of the patient cohort.

### Comparison of SCRC and MCRC to PCRC

Age did not differ significantly between patients with PCRC, SCRC, and MCRC. There was a higher proportion of men in the SCRC group (68% vs. 57% for PCRC; *p*=0.027). In addition, complete tumor obstruction (manifest ileus) was found to be more frequent in patients with SCRC (9% vs. 5% in PCRC; *p*=0.017). Rectal cancer was present in 41% of the patients with PCRC, compared to only 28% (*p*<0.001) for SCRC and 33% for MCRC (*p*<0.001). In 29% of the patients with SCRC, the synchronous multiple tumors were not in the same segment, while in 71% of patients with SCRC, all tumors were located within the same segment (proximal colon/distal colon/rectum). The maximum tumor size of MCRC was significantly smaller (3.6cm vs. 4.0cm for PCRC; *p*<0.001). Patients with MCRC more frequently underwent multivisceral resection of the omentum, small bowel, abdominal wall, bladder, ureter, ovar/adnexes, prostate, pancreas, and others (31% vs. 19% for PCRC; *p*=0.002). Regarding histopathology, the R0 rate was significantly higher in MCRC patients (86% vs. 79% in PCRC; *p*=0.030). High-grade tumors (G3 or G4) occurred more frequently in SCRC (43% vs. 31% for PCRC; *p*=0.008). Although only available in a very limited number of patients, no significant differences occurred in microsatellite instability, KRAS mutations, or BRAF mutations. Of the patients with MCRC, most initial resections were performed at another institution. Thus, only limited information was available regarding the time period between both tumor occurrences, staging, and treatment of the initial tumor. There was no evidence for hereditary forms of SCRC and MCRC in the analyzed cohort, respectively. See Table 2 for characteristics of patients stratified for PCRC, SCRC, and MCRC.

**Table 2:** Characteristics of singular primary colorectal cancers (PCRC), multiple synchronous colorectal cancers (SCRC), and multiple metachronous colorectal cancers (MCRC)

	Primary unifocal CRCs (PCRC)		Multiple synchr. CRCs (SCRC)		*p* (PCRC vs SCRC)	Metachr. CRC (MCRC)		*p* (PCRC vs MCRC)
	*n*=3506	%	*n*=103	%		*n*=105	%	
Patient age								
Years (median)	64	100	68	100	*0.009*	68	100	*0.062*
Gender								
Men	1999	57	70	68	*0.027*	66	63	*0.233*
Women	1507	43	33	32		39	37	
Colon versus rectum (main tumor)								
Colon	2060	59	70	68	*0.061*	73	70	*0.027*
Rectum	1446	41	33	32		32	30	
Localization								
Multiple segments	0	0	30	29	*<0.001*	0	0	*<0.001*
Right hemicolon	1069	31	29	28		36	37	
Left hemicolon	968	28	15	15		30	30	
Rectum	1429	41	29	28		33	33	
Missing	40		0			6		
Preoperative ileus								
No ileus	2995	89	92	90	*0.017*	88	88	*0.956*
Subileus	192	6	1	1		7	7	
Manifest ileus	164	5	9	9		5	5	
Missing	155		1			5		
Concomitant diseases								
No	1497	46	27	28	*0.003*	23	23	*<0.001*
Yes	1747	54	68	72		76	77	
Missing	262		8			6		
Tumor size								
Centimeter (median)	4.0	100	4.5	100	*0.054*	3.6	100	*<0.001*
Missing	273		5			8		
Multivisceral surgery								
No	2666	81	79	81	*0.979*	69	68	*0.002*
Yes	640	19	18	19		33	32	
Missing	200		6			3		
Tumor status								
T0	62	2	2	2	*0.173*	1	1	*0.687*
Tis	21	1	0	0		2	2	
T1	334	10	5	5		10	10	
T2	591	18	11	11		21	21	
T3	1737	52	62	62		50	50	
T4	580	17	20	20		16	16	
Missing	181		3			5		
Nodal status								
N0	1832	56	47	47	*0.301*	64	65	*0.126*
N1	740	22	24	24		22	23	
N2	722	22	29	29		12	12	
Missing	212		3			7		
Metastasis status								
M0	2455	76	73	74	*0.670*	77	83	*0.111*
M1	792	24	26	26		16	17	
Missing	259		4			12		
Stage								
0/Tis	68	2	2	2	*0.262*	1	1	*0.153*
I	716	22	14	14		23	25	
II	843	26	27	27		30	33	
III	809	25	30	31		21	23	
IV	792	25	26	26		16	18	
Missing	278		4			14		
Grading								
G1/2	2254	69	57	57	*0.008*	70	71	*0.787*
G3/4	992	31	43	43		29	29	
Missing	260		3			6		
Lymphatic invasion								
L0	2451	74	67	68	*0.206*	81	79	*0.202*
L1	843	26	32	32		21	21	
Missing	212		4			3		
Vascular invasion								
V0	3002	91	91	92	*0.622*	94	93	*0.497*
V1	289	9	8	8		7	7	
Missing	215		4			4		
R status								
R0	2621	79	75	77	*0.875*	85	86	*0.030*
R1	99	3	2	2		6	6	
R2	599	18	20	21		8	8	
Missing/Rx	187		6			6		
Microsatellite status								
MSS	257	75	5	62	*0.617*	8	89	*0.604*
MSI-H	87	25	3	38		1	11	
Not performed	3162		95			96		
KRAS status								
Wildtype	196	66	5	62	*0.949*	5	50	*0.530*
Mutated	103	34	3	38		5	50	
Not performed	3207		95			95		
BRAF status								
Wildtype	228	85	8	100	*0.485*	7	100	*0.537*
Mutated	41	15	0	0		0	0	
Not performed	3237		95			98		
Tumor perforation								
No	2381	94	82	98	*0.037*	81	94	*0.104*
Yes	164	6	2	2		5	6	
Missing	961		19			19		
Tumor histology								
Classical adenocarcinoma	2899	86	92	91	*0.414*	88	85	*0.508*
Mucinous adenocarcinoma	364	11	6	6		12	12	
Signet-ring cell carcinoma	29	1	1	1		2	2	
Others	62	2	2	2		1	1	
Missing	152		2			2		
CEA (ng/ml, median)								
ng/ml (Median)	4.0	100	5.0	100	*0.319*	3.0	100	*0.166*
Not performed	2332		77			82		
Tumor recurrence								
No	1772	59	42	49	*0.476*	60	76	*0.002*
Yes	1241	41	44	51		19	24	
Lost of follow-up	493		17			26		
Alive status								
Alive	1371	46	31	38	*0.189*	55	69	*<0.001*
Death cancer-related	1090	37	37	56		10	13	
Death postoperative	108	4	1	1		3	4	
Death other causes	374	13	12	15		11	14	
Lost of follow-up	563		22			26		

The *p*-values refer to the comparison of PCRC to SCRC, respective PCRC to MCRC, as indicated

### Survival

**Fig. 1:**
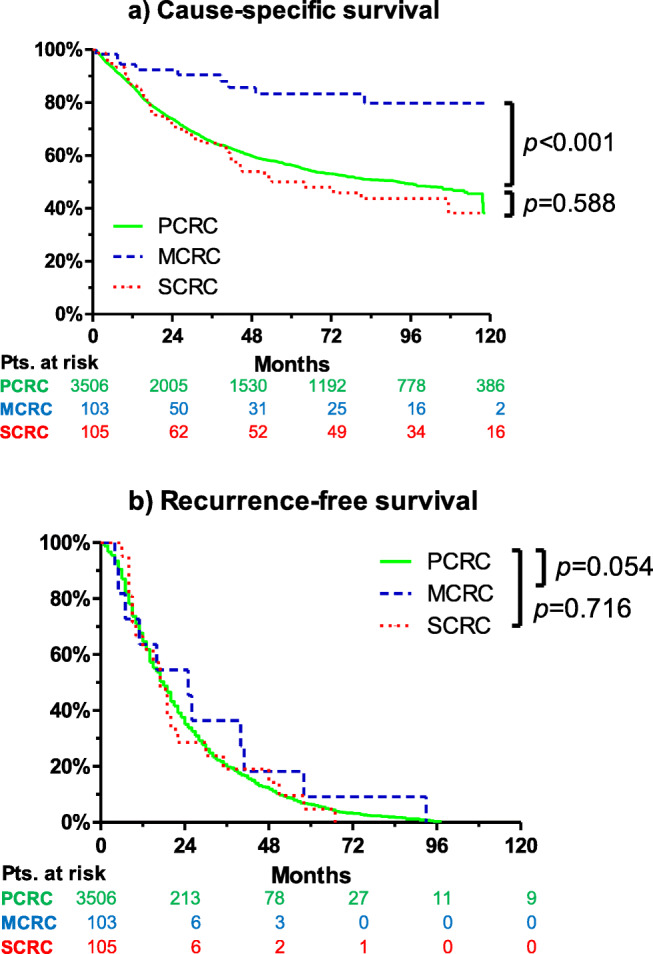
Cause-specific survival (**a**) and recurrence-free survival (**b**) of patients with primary colorectal cancer (PCRC), multiple synchronous colorectal cancer (SCRC), and multiple metachronous colorectal cancer (MCRC). Kaplan-Meier curves showed a clear survival benefit for MCRC, compared to PCRC and SCRC. In contrast to survival data, for recurrence free survival, a considerable reduced number of validated patient data were available for SCRC and MCRC. Thus, the informative value of Kaplan-Meier curve (**b**) may be limited

**Fig. 2:**
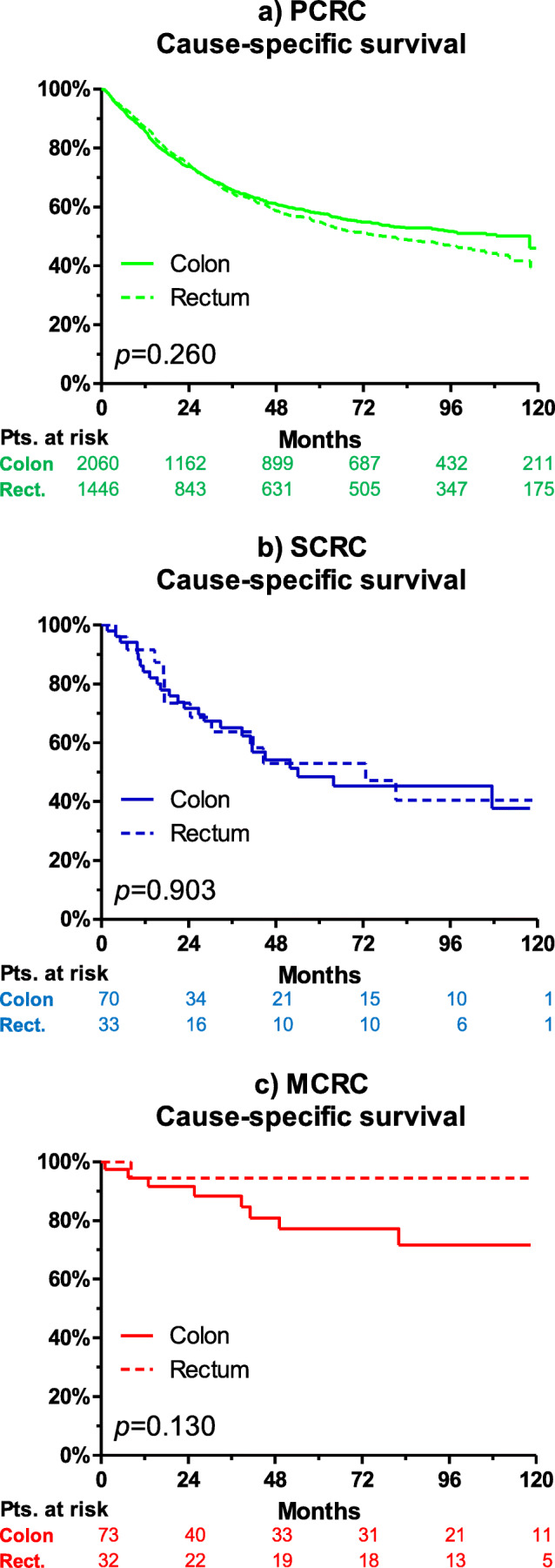
Cause-specific survival of patients with (**a**) PCRC, (**b**) SCRC, and (**c**) MCRC, stratified by colon cancer versus rectal cancer. No difference was observed between colon and rectal cancer for each spatial tumor manifestation

While tumor recurrence rates during follow-up was comparable for PCRC (41%) and SCRC (51%; *p*=0.476), MCRC had a significantly lower rate of recurrence (24%, *p*=0.002). These results are in line with the Kaplan-Meier analyses, where MCRC had a significantly better cause-specific survival compared to PCRC and SCRC. Interestingly, no significant differences were observed between the groups for recurrence-free survival (see Fig. 1 for survival rates stratified by PCRC, SCRC, and MCRC). Five-year cause-specific survival rates were 63±1% for PCRC, 62±6% for SCRC (*p*=0.588), and 88±4% for MCRC (*p*<0.001). Median cause-specific survival was not reached for PCRC and MCRC. For SCRC, median cause-specific survival was 63.9 months (95% CI 10 to 118). No significant differences in survival between patients with colon cancer and rectal cancer were observed among the PCRC (*p*=0.260), SCRC (*p*=0.903), and MCRC groups (*p*=0.130). Figure 2 shows the cause-specific survival of patients with PCRC, SCRC, and MCRC, stratified by colon cancer versus rectal cancer.

### Prognosis of SCRC and MCRC

Compared to PCRC, patients with SCRC had a slightly, but not significantly, increased hazard ratio for cause-specific death (*p*=0.057; HR 1.38; 95% CI 0.99–1.91). In contrast, patients with MCRC had a significantly better prognosis (*p*<0.001; HR 0.27; 95% CI 0.14–0.52). Further, factors associated with a reduced prognosis upon univariable analysis are listed below: preoperative manifest ileus (*p*<0.001; HR 2.40; 95% CI 1.91–3.03), extensive tumor size (*p*<0.001; per centimeter: HR 1.14; 95% CI 1.11–1.16), multivisceral surgery (*p*<0.001; HR 1.65; 95% CI 1.44–1.90), high T, N, M, and UICC stage (*p*<0.001 for each), G3/4 vs. G1/2 (*p*<0.001; HR 2.20; 95% CI 1.95–2.48), L1 (lymphatic invasion; *p*<0.001; HR 3.28; 95% CI 2.90–3.70), V1 (vascular invasion; *p*<0.001; HR 3.96; 95% CI 3.36–4.67), non-R0 (*p*<0.001 for R1; HR 5.94; 95% CI 4.53–7.79), microsatellite stable tumors (*p*=0.011; HR 2.94; 95% CI 1.28–6.67), KRAS mutated tumors (*p*=0.011; HR 1.93; 95% CI 1.17–3.19), tumor perforation (*p*<0.001; HR 2.61; 95% CI 2.02–3.38), histological tumor type other than classical adenocarcinoma (*p*<0.001 for mucinous adenocarcinoma, signet-ring cell carcinoma, and other rare types, respectively), and CEA (*p*<0.001; HR 1.00 per 1ng/ml; 95% CI 1.00–>1.00). A multivariable analysis was performed, which included all prognostic factors of the univariable analysis. Here, only tumor size, stable microsatellites, histological tumor type, and CEA remained independent significant predictors of survival (not shown). However, due to the reduced number of events (low absolute number of cause-specific deaths in the SCRC and MCRC group), another more robust multivariable analysis was performed, which only included the clinically most relevant factors. See Table 3 for the multivariable analysis. Here, MCRC was again identified as an independent favorable prognostic factor regarding cause-specific survival (*p*=0.005; HR 0.25; 95% CI 0.09–0.66). In accordance to the above-mentioned data, other poor prognostic factors upon this adjusted multivariable analysis were tumor length, UICC stage, R status other than R0, tumor histology, and CEA levels.

**Table 3:** Multivariable analysis as described in the text

	*p*	HR	95% CI	
			Lower	Upper
Tumor type				
Primary unifocal CRC (PCRC)	*0.010*	1		
Multiple synchronous CRC (SCRC)	*0.264*	1.28	0.83	1.98
Metachronous CRC (MCRC)	*0.005*	0.25	0.09	0.66
Tumor size				
Centimeter (continuously)	*0.016*	1.05	1.01	1.09
Stage				
0/Tis	*<0.001*	1		
I	*0.787*	0.82	0.20	3.45
II	*0.315*	2.06	0.51	8.37
III	*0.019*	5.34	1.32	21.55
IV	*0.002*	9.23	2.25	37.90
Grading				
G1/2		1		
G3/4	*0.096*	1.15	0.98	1.35
R status				
R0	*<0.001*	1		
R1	*<0.001*	3.04	2.10	4.41
R2	*<0.001*	4.45	3.33	5.94
Tumor histology				
Classical adenocarcinoma	*<0.001*	1		
Mucinous adenocarcinoma	*0.040*	1.28	1.01	1.63
Signet-ring cell carcinoma	*<0.001*	4.04	2.41	6.77
Other rare types	*<0.001*	2.48	1.51	4.09
CEA (ng/ml, median)				
ng/ml (continuously)	*<0.001*	>1.00	1.00	>1.00

After correction for tumor stage, R status, histology, and CEA, the tumor type was still an independent predictor of case-specific survival, with better prognosis for MCRC. *HR* hazard ratio, *95% CI* 95% confidence interval

### Subgroups of special interest

**Fig. 3:**
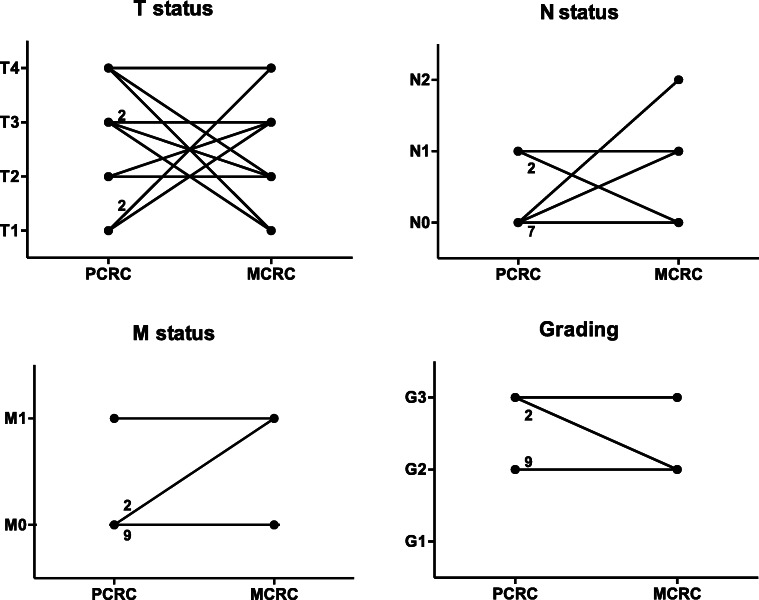
Trend analysis of the twelve patients who underwent resection of their initial primary colorectal cancer (PCRC) as well as for their metachronous colorectal cancer (MCRC) years later at our institution. Every line that illustrates more than just one patient is provided with the respective numbers of patients in this group. A relationship was identified neither for the TNM classification nor for the tumor grading. Regarding the M1-PCRC shown in the left lower panel, one patient with a primary carcinoma of the sigmoid and liver metastasis underwent combined resection (R0) but developed metachronous cancer of the ascending colon with a limited carcinomatosis peritonei 4 years later. He was alive without recurrence 2 years after the last operation

Of all documented patients, only twelve underwent resection at our department for both, the initial PCRC as well as for the MCRC at a later time point, without a clear risk profile regarding comorbidities or other factors. Eight of these were male, and four were female. The median age at the time of the first tumor manifestation was 68 years. Notably, the MCRC occurred after a median time of 9 years (range 3 to 19 years) after the PCRC. Regarding TNM classification and tumor grading, no correlation was identified between the PCRC and the corresponding MCRC. See Fig. 3 for trend analysis of patients resected for PCRC, followed by MCRC resection. All patients underwent another tumor resection, and none of these patients developed a third tumor manifestation during follow-up.

In total, only two patients were detected, who had a manifestation of metachronous colorectal cancer (MCRC) that presented as synchronous multiple colorectal cancer (SCRC). In specific, a 77-year-old male patient with rectal cancer diagnosed and resected about 35 years ago had now two tumor manifestations within the ascending colon. Additionally, a 91-year-old female patient who had a T3N0M0 rectal cancer 5 years ago presented now with a tumor manifestation in the transverse colon, and another tumor manifestation in the rectum, which were not classified as a local recurrence. The first patient did not have recurrence of the disease after the second tumor resection until the last follow-up 90 months after surgery. However, the second patient with rectal cancer developed a tumor recurrence and finally died from systemic metastasis. For clarity and to avoid interference with the results, those two patients who could not clearly classifiable to MCRC or SCRC were not included in the further analysis, apart from their description in this paragraph.

## Discussion

This study considerably enlarges the current knowledge of rare types of multiple colorectal cancers by providing a comprehensive analysis of more than 3500 patients with PCRC, SCRC, or MCRC, including clinical, histopathological, molecular genetics, and follow-up data. By analyzing a large collective over a period of 37 years, we did not detect any survival difference for patients with multiple synchronous colorectal cancers (SCRC) compared to patients with classical primary unifocal colorectal cancer (PCRC). However, patients with metachronous colorectal cancer (MCRC) had an improved prognosis, which was an independent factor in multivariable analysis.

Of all analyzed CRC patients, 2.8% had SCRC and MCRC. These findings as well as other baseline characteristics are in line with previously reported results, underlining the general validity of our data [3, 5, 7, 16]. Of course, a limitation of the analysis is the retrospective and unicentric study design over a very long time, potentially leading to selection or treatment bias since the majority of initial cancers were not treated at the study institution. However, as no study intervention was performed and considering the high number of included patients, observational approach is deemed most appropriate in this context. Further, patients with HNPCC or APC may have been misinterpreted as sporadic SCRC or MCRC in this analysis. However, as determined by patient history, clinical assessment, genetic analysis in a part of the patients, and thorough follow-up, hereditary forms of colorectal cancer were excluded as far as possible in this study.

The improved survival for patients with MCRC compared to PCRC was the most relevant finding of our study and remains an object of discussion. This is the most comprehensive analysis hitherto existing, which reports not only on the incidence but also long-term survival of patients with sporadic metachronous colorectal cancer [6, 17]. Possible explanations for an improved survival of MCRC patients are a positive selection of patients in this surgical cohort with locally restricted and resectable manifestations of the metachronous carcinoma. Also, stricter adherence to follow-up regimens after the second tumor manifestation could have improved early detection and treatment of secondary colorectal cancers and a higher patient compliance. Accordingly, lower T stages were found in the MCRC group, although not reaching a significant range. However, MCRC remained an independent favorable prognostic factor in the multivariable analysis (Table 3). Compared to the rectum, the tumors were more frequently located in the colon in patients with MCRC (70%) than in patients with PCRC (59%; see Table 2); however, tumor location (colon vs. rectum) was not a prognostic factor in this patient cohort (*p*=0.320; Table 1). This would indicate other, potentially molecular genetic factors responsible for the improved prognosis of MCRC.

Observational studies report an elevated risk for MCRC after initial SCRC (HR 3-6) [6, 7]. Of note, two patients with SCRC were identified during data collection for this study, who developed a MCRC during follow-up. As reported above, these patients were not included within the analysis in order to prevent any possible selection bias. However, the estimated number of unreported cases is considerably higher due to admission to another hospital or misinterpretation as tumor recurrence.

In the subgroup of patients who were documented for both, resection of their PCRC and SCRC, a median interval of 9 years between the two tumor manifestations was observed. Earlier, Jayasekara et al. reported a mean interval of 4 years; however, the mean follow-up of our study was longer than the mean follow-up by Jayasekara et al. Thus, metachronous tumors after a longer interval may have been missed in the latter study. This finding raises the question for longer follow-up intervals than the usually performed 5 years, at least for selected patients at high risk for a MCRC (see Table 2).

## Conclusion

Development and survival differences for PCRC, SCRC, and MCRC still cannot be fully explained. Current studies address factors arising from genetics (HNPCC, APC, IBD) [3, 5], environment (hypertension, hypoalbuminemia) [4, 6], and iatrogenic issues (tumor seeding during colonoscopy) [18].

Since this study identified metachronous colorectal cancers to develop even many years after the initial tumor, this exploratory study suggests that a programmed long-term follow-up may be the only pragmatic recommendation for patients with sporadic multiple colorectal cancers at present.

There are no large studies with validated prognostic or predictive factors available up to now. The retrospective study reported here may be prone to bias; thus further genetic and multicenter long-term studies are required.

## Supplementary information

ESM 1 (DOCX 30 kb)

## Declarations

This study was performed in line with the principles of the Declaration of Helsinki. Approval was granted by the Ethics Committee of the Technical University of Munich, as stated in the methods part. The study report follows the STROBE statement for observational studies.
